# The antibody drug conjugate VLS-101 targeting ROR1 is effective in CAR T-resistant mantle cell lymphoma

**DOI:** 10.1186/s13045-021-01143-w

**Published:** 2021-08-28

**Authors:** Vivian Changying Jiang, Yang Liu, Alexa Jordan, Joseph McIntosh, Yijing Li, Yuxuan Che, Katti A. Jessen, Brian J. Lannutti, Michael Wang

**Affiliations:** 1 Department of Lymphoma and Myeloma, The University of Texas, MD Anderson Cancer Center, 1515 Holcombe Blvd., Houston, TX USA; 2 VelosBio Inc., San Diego, CA USA; 3 Department of Stem Cell Transplantation and Cellular Therapy, The University of Texas MD Anderson Cancer Center, Houston, TX USA

**Keywords:** Mantle cell lymphoma, ROR1, VLS-101, CAR T resistance

## Abstract

Mantle cell lymphoma (MCL) is a rare, aggressive and incurable subtype of non-Hodgkin’s B-cell lymphoma. The principal barrier is frequent clinical relapse to multiple lines of therapies, including new FDA-approved biologics and cell therapy. Brexucabtagene autoleucel, the first and only FDA approved chimeric antigen receptor (CAR) T product in MCL, demonstrated unprecedented efficacy in overcoming resistance to Bruton’s tyrosine kinase inhibitors. However, relapses have inevitably occurred and once relapsed these patients display a very poor clinical outcome. Currently, there is no optional therapy specifically designed for these patients. The development of tailored and more efficacious therapies is therefore critical and represents a new medical need. We found that while the receptor tyrosine kinase-like orphan receptor 1 (ROR1) is expressed across most of the MCL cells, it is significantly elevated in CAR T-relapsed MCL tumors. To see whether this aberrant ROR1 expression contributed to CAR T resistance, we targeted ROR1 using VLS-101, a monomethyl auristatin E conjugated anti-ROR1 antibody. VLS-101 showed potent anti-MCL activity in vitro in ROR1-expressing MCL cell lines and ex vivo in primary patient samples. Importantly, VLS-101 safely induced tumor regression in PDX models resistant to CAR T-cell therapy, ibrutinib and/or venetoclax. These data advocate for targeting ROR1 as a viable approach in the treatment of ROR1-positive MCL tumors, especially those with failure to prior therapies. These data also provide strong evidence for future enrollment of post-CD19 CAR T-cell relapsed MCL patients in a first in-human phase 1b VLS-101 trial. The upcoming testing in a clinical setting will provide important insights on this new therapeutic development aiming to overcome the CAR T resistance via targeting ROR1, which is a rising unmet clinical need in MCL.

**Supplementary Information:**

The online version contains supplementary material available at 10.1186/s13045-021-01143-w.

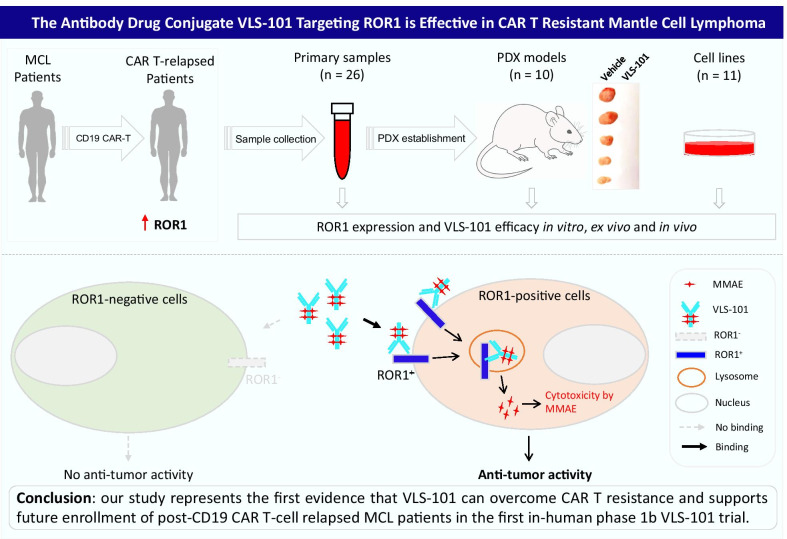

## To the Editor

Brexucabtagene autoleucel (BA) [1], is the first and only chimeric antigen receptor (CAR) T product approved by FDA for relapsed or refractory (R/R) mantle cell lymphoma (MCL) based on its recently reported safety and unprecedented efficacy (ORR 93%) [2]. Unfortunately, relapses do occur; relapsed patients display a median survival of only 4.1 months[3]. This rapidly growing clinical challenge warrants mechanistic decoding of CAR T resistance and new therapeutic developments to overcome it.

Receptor tyrosine kinase-like orphan receptor 1 (ROR1) is an embryo-oncogenic transmembrane receptor with tightly controlled expression in normal tissues [4]. Its aberrant expression was shown in many cancer types including MCL [4, 5]. High ROR1 expression associates with aggressive disease, poor survival and therapeutic resistance [6, 7]. Interestingly, ROR1 forms a functional complex with CD19 on cell surface that activates various signaling pathways to promote MCL cell proliferation in a B-cell receptor/BTK signaling-independent manner [8].

**Fig. 1:**
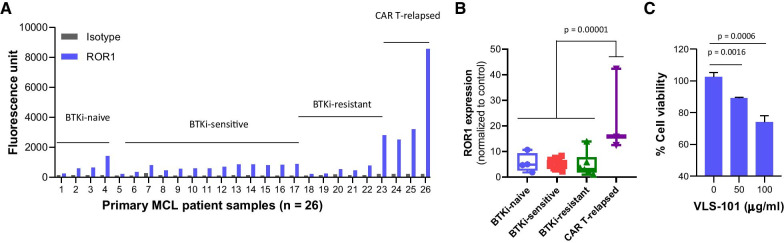
ROR1 expression and targeting in primary MCL patient samples, including CAR T-resistant MCL patient cells. (**a**) ROR1 expression detected by flow cytometry in primary MCL patient samples (*n* = 26), classified into four groups: BTKi-naïve (*n* = 4), BTKi-sensitive (*n* = 13), BTKi-resistant (*n* = 6) and CAR T-relapsed (*n* = 3). (**b**) Cell surface ROR1 expression of the four groups in (**a**) normalized to controls. (**c**) Dose-dependent inhibition of cell viability by MMAE-conjugated ROR1 antibody VLS-101. ROR1 targeting by VLS-101 is shown in a CAR T-relapsed MCL patient sample. Student *t* test was used to calculate statistical significance

We detected ROR1 expression in 92% (24/26) MCL samples collected (non-preferentially) by either apheresis or by excisional biopsy as per physician’s directions (Fig. 1a). ROR1 was positive in all patient-derived xenograft (PDX) models (*n* = 10) and MCL cell lines (9/11, 82%) (Additional file 1: Figure S1A and S2A). Interestingly, ROR1 expression was highest in BA-relapsed samples (*n* = 3) (*p* = 0.00001) (Fig. 1b), suggesting that ROR1 may be a driver of BA resistance in MCL and targeting ROR1 may overcome it.

UC-961 is a first-in-class humanized monoclonal specific anti-ROR1 antibody, shown to be safe in preclinical and clinical trials [9, 10]. To increase its anti-tumor activity, VLS-101 was created by conjugating UC-961 to monomethyl auristatin E (MMAE) via a cleavable linker [11]. VLS-101 retains UC-961 specificity in targeting ROR1 on cell surface and induces a target-antibody complex internalization which facilitates MMAE release in the lysosome to kill cells [12].

Ex vivo treatment with VLS-101 induced dose-dependent cytotoxicity in ROR1^+^ primary MCL cells collected from a BA-relapsed patient (Fig. 1c). Consistently, VLS-101 treatment resulted in potent cytotoxicity in ROR1^+^ but not in ROR1^−^ MCL cell lines at a low IC_50_ range (4.0–23.3 µg/ml, at 72 h) and in a dose- and time-dependent manner (Additional file 1: Figure S1B–E). VLS-101 induced cell apoptosis and cell cycle arrest at G2/M (Additional file 1: Figure S1F-G) likely due to intracellular release of MMAE upon ROR1 engagement and internalization [12].

**Fig. 2:**
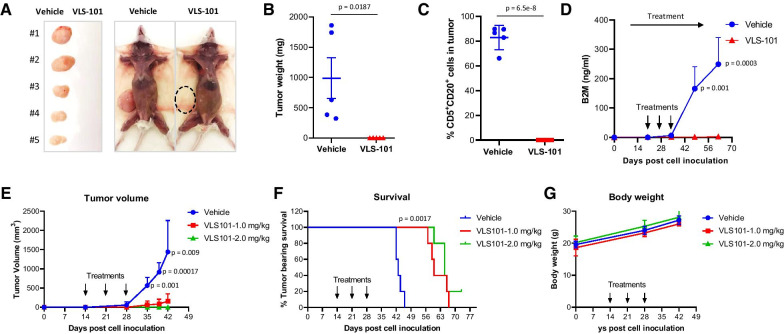
VLS-101 is potent in targeting ROR1-expressing PDX models with dual resistance to ibrutinib and CAR T. A PDX model was established from one of the ibrutinib-CAR T dual resistant MCL patient in immunodeficient NSG mice. (**a–d**) Freshly isolated PDX cells from previous generation were inoculated subcutaneously into NSG mice. When tumors became palpable (at day 20 post cell inoculation), the mice (*n* = 5) were treated intravenously (IV) with vehicle (*n* = 5) or VLS-101 (*n* = 5) at 2.5 mg/kg (QW × 3). When the tumors in the vehicle groups reached 15 mm in diameter, the subcutaneous tumors were dissected, imaged (**a**) and weighted (**b**). The percentage of MCL cells in the subcutaneous tumors were detected by flow cytometry with fluorescence conjugated CD5 and CD20 antibodies (**c**). B2M levels were detected by B2M ELISA kits using the mouse serum collected every two weeks (**d**). (**e–g**) The same model was used to pass on to the next generation and the mice were treated with VLS-101 at lower doses 1 and 2 mg/kg (QW × 3, IV) (*n* = 5 per group) when tumors were palpable (at day 14 post cell inoculation). Tumor volume (**e**), mice survival (**f**), and body weight (**g**) were monitored and plotted. We used the 15 mm tumor size as humane end points for animal survival analysis. Student *t* test was used to calculate statistical significance

To determine whether VLS-101 can overcome therapeutic resistance, patient-derived xenograft (PDX) models were successfully established in immunodeficient NSG mice. Importantly, ROR1 expression retains from patients to PDX (p = 0.76, Additional file 1: Figure S2B). VLS-101 treatment at 2.5 mg/kg (weekly × 3, intravenously) eliminated subcutaneous tumor growth of BA-resistant PDX model (PDX-1) (Fig. 2a–c). This was confirmed by the production of serum beta-2-microglobulin (B2M), a systemic tumor load marker (Fig. 2d). We repeated the experiment with lower doses to further check its potency; the tumors were barely palpable (mean = 160 mm^3^) and completely regressed when treated with VLS-101 at 1 and 2 mg/kg, respectively. In contrast, vehicle-treated tumors reached a mean volume of 1440 mm^3^ (Fig. 2e). In addition, VLS-101 treatment significantly prolonged tumor bearing mouse survival (60 and 65 days, respectively), compared to vehicle-treated group (43 days, *p* = 0.0017) (Fig. 2f). Importantly, VLS-101 treatment at 1, 2 and 2.5 mg/kg was well-tolerated as determined by gross observation and body weight measurements (Fig. 2g). Consistent VLS-101 efficacy was also observed in the disseminated PDX model (PDX-2) derived from an ibrutinib-venetoclax dual-resistant patient tumor with positive ROR1 expression (Additional file 1: Figure S2B). Treatment with VLS-101 at 2.5 mg/kg eradicated the tumors in the spleen (*p* = 0.0001), and significantly inhibited tumor growth in the liver (*p* = 0.002), compared to vehicle-treated group (Additional file 1: Figure S3A–C).

Collectively, we demonstrated that BA-relapsed patients express aberrantly higher ROR1 compared to other MCL tumors and that VLS-101 is highly effective in resistant MCL preclinical models. Most importantly, VLS-101 treatment was safe and efficient in eradicating BA-resistant tumors in subcutaneous PDX models and ibrutinib-venetoclax resistant tumors in disseminated PDX models. At the 2020 American Hematology Society annual meeting, VLS-101 was reported to be safe and effective in overcoming ibrutinib resistance (ORR 47%) in a first in-human phase 1b trial [13]. A cohort expansion of post-CD19 CAR T-cell relapsed MCL patients is being considered based on our study. Translational and mechanistic studies are currently ongoing to understand why post-CD19 CAR T-cell relapsed tumors are hypersensitive to VLS-101 targeting embryo-oncogenic ROR1 in MCL.

## Supplementary Information

**Additional file 1**. Supplemental Figures.

## Abbreviations

BA: Brexucabtagene autoleucel
B2M: Beta-2-microglobulinBTK: Bruton’s tyrosine kinase
BTKi: BTK inhibitor
CAR: Chimeric antigen receptor
ROR1: Receptor tyrosine kinase-like orphan receptor 1
MCL: Mantle cell lymphoma
MMAE: Monomethyl auristatin E
PDX: Patient-derived xenograft

## Declarations

### Availability of data and materials

All data generated or analyzed during this study are included in this published article and its supplementary information files.
