# Supplementary material for: The antibody drug conjugate VLS-101 targeting ROR1 is effective in CAR T-resistant mantle cell lymphoma
Source: J Hematol Oncol. 2021 Aug 28;14:132. doi: 10.1186/s13045-021-01143-w (PMC8400406; doi:10.1186/s13045-021-01143-w)
Supplement: Supplementary file 1 — Additional file 1. Supplemental Figures. [file 13045_2021_1143_MOESM1_ESM.pdf]

Supplementary Figure S1

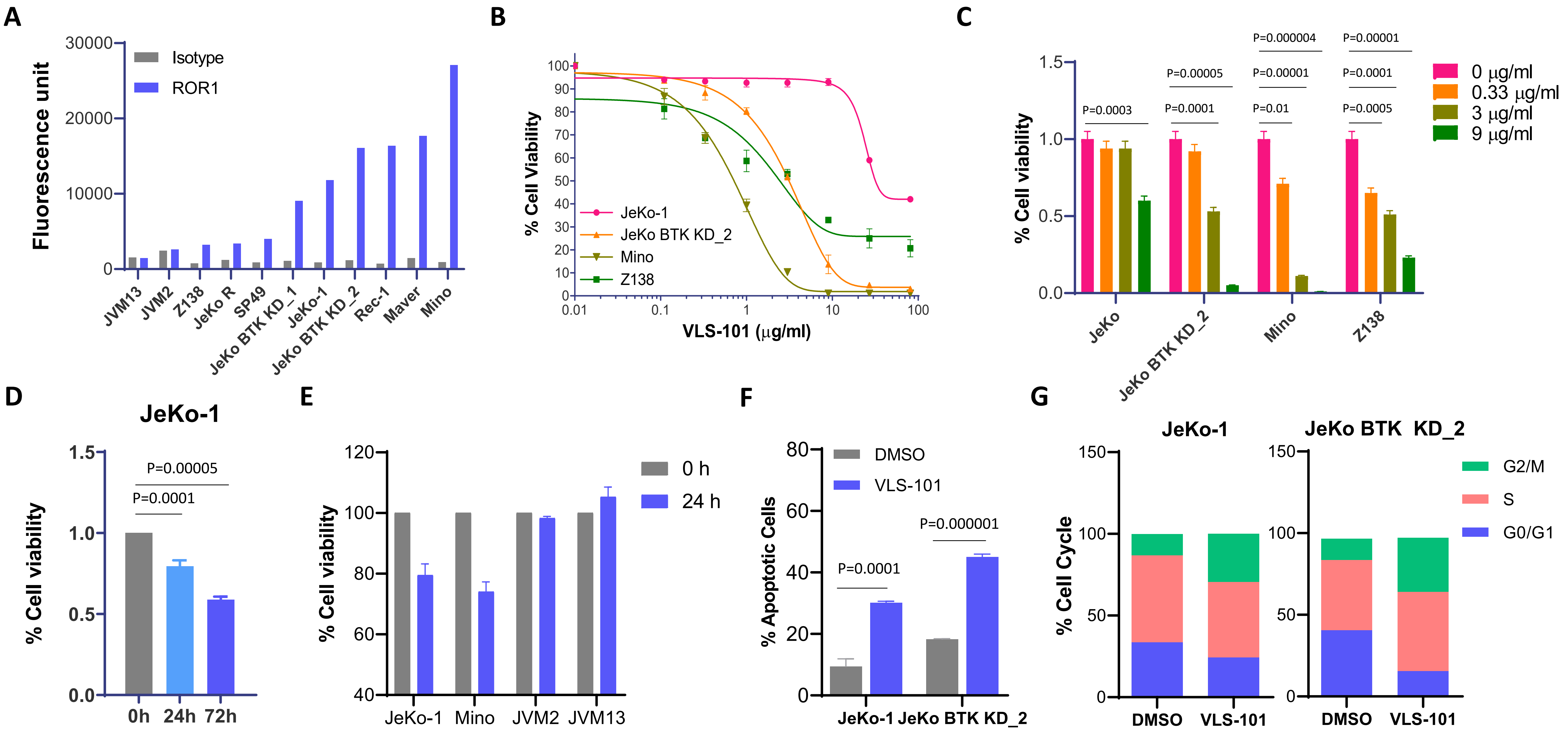

Supplementary Figure S2

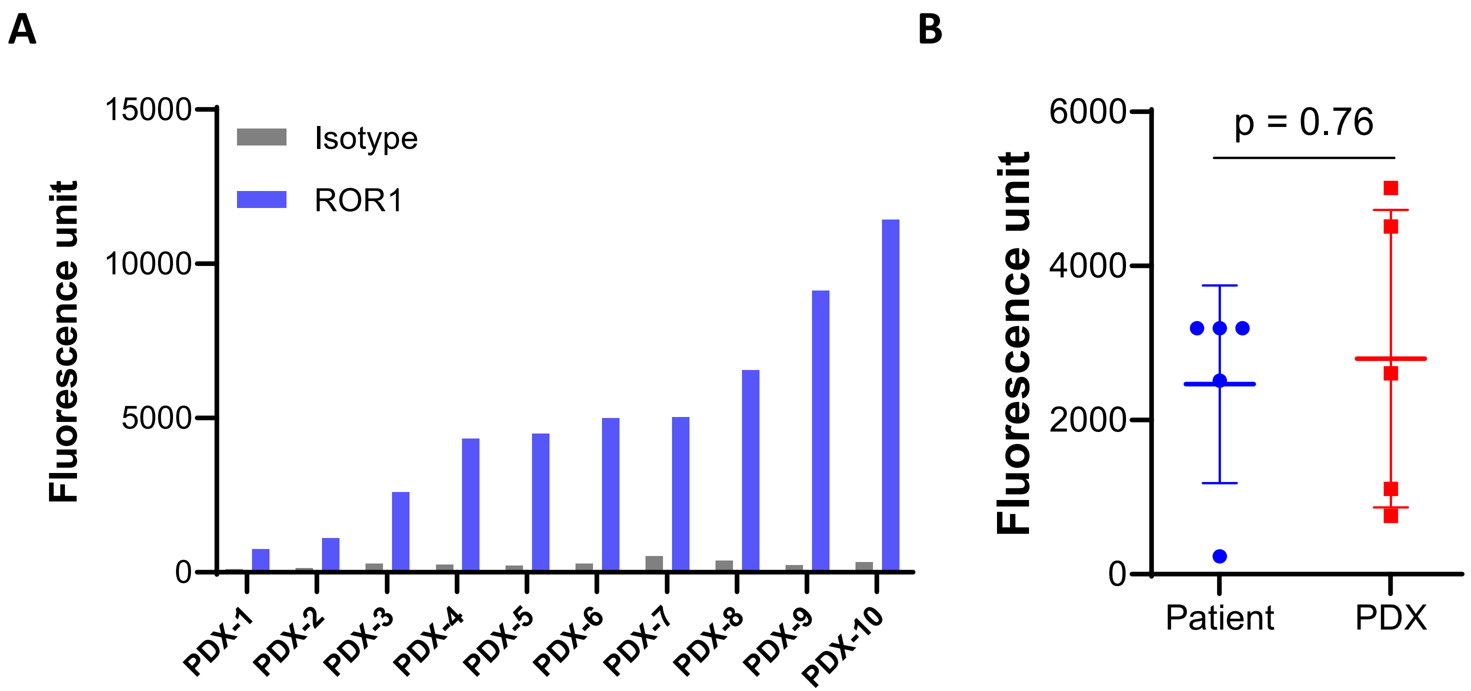

Supplementary Figure S3

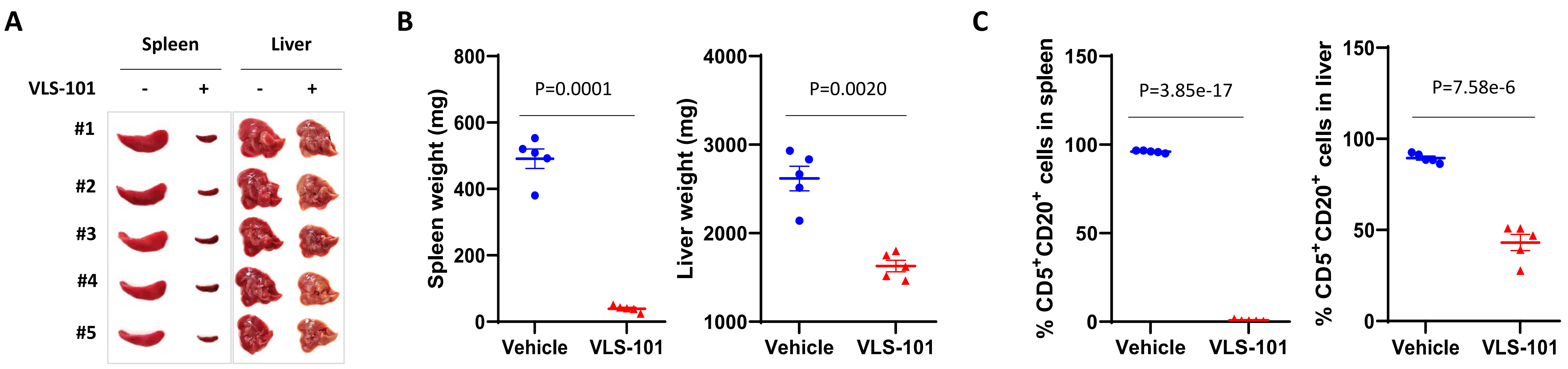

Supplementary Figures

Figure S1. ROR1 expression and ROR1 targeting in MCL cell lines. (**A**) ROR1 expression detected by flow cytometry in MCL cell lines (n = 11). (**B**) *In vitro* efficacy of VLS-101 in 4 representative MCL cell lines, JeKo-1, JeKo-BTK-KD, Mino and Z138 at 72 h post treatment with VLS-101. (**C**) Dose-dependent inhibition of cell viability by VLS-101 at 24 h post treatment in MCL cell lines. (**D**) Time-dependent inhibition of cell viability by VLS-101 treatment in JeKo-1 cells. (**E**) ROR1 expression dependent cell viability inhibition at 24 h post treatment. Note the lack of VLS-101 inhibitory effect in ROR1-negative cells (JVM2 and JVM13). (**F-G**) Cell apoptosis (**F**) and cell cycle arrest (**G**) induction by VLS-101 at 24 h post treatment in ibrutinib-sensitive (JeKo-1) and resistant (JeKo BTK KD_2) cell lines. Student *t* test was used to calculate statistical significance.

Figure S2. ROR1 expression in PDX models and their parental patient samples. (**A**) ROR1 expression detected by flow cytometry in MCL PDX models (n = 10). (**B**) Comparison of ROR1 expression levels between parental clinical samples and PDX models using flow cytometry (n = 5).

Figure S3. VLS-101 is potent in targeting MCL cells in ROR1-expressing PDX models with dual resistance to ibrutinib and venetoclax. A PDX model was established from an ibrutinib-venetoclax dual resistant MCL patient tumor using a multi-generational expansion in immunodeficient NSG mice. Fresh isolated PDX cells from the spleen of previous mouse generation were inoculated intravenously into NSG mice. At 2 weeks post cell inoculation, the mice were treated intravenously with vehicle (n = 5) or VLS-101 (n = 5) at 2.5 mg/kg (QW x 3). (**A-C**) At 8 weeks post cell inoculation, the mouse spleen (left panel) and liver (right panel) were dissected, imaged (**A**) and weighted (**B**). The percentage of MCL cells in the spleen (left panel) and liver (right panel) were detected by flow cytometry with fluorescence conjugated CD5 and CD20 antibodies (**C**). Student *t* test was used to calculate statistical significance.
